# Upfront Radiosurgery for Treatment of Symptomatic Obstructive Hydrocephalus due to Brain Tumors

**DOI:** 10.7759/cureus.29129

**Published:** 2022-09-13

**Authors:** Alejandra Moreira, Alejandra Rodezno, David Santos, Adriana Telles, Juliana Ramirez, Eduardo E Lovo

**Affiliations:** 1 Neurosurgery, International Cancer Center, San Salvador, SLV; 2 Radiosurgery, International Cancer Center, San Salvador, SLV; 3 Radiosurgery, Centro de Radiocirugia Robotica, San Jose, CRI

**Keywords:** two-stage radiosurgery, radiosurgery, metastases, primary tumor, obstructive hydrocephalus

## Abstract

Introduction

Hydrocephalus is a build-up of cerebrospinal fluid (CSF) in the brain and is characterized by abnormal dilatation of the cerebral ventricles. Patients can be either asymptomatic, have symptoms related to primary tumors, or have hydrocephalus-related symptoms. Generally, symptomatic patients are candidates for ventriculoperitoneal (VP) shunt placement to reduce acute symptoms. Little evidence exists regarding the resolution of symptomatic hydrocephalus secondary to brain tumors using stereotactic radiosurgery (SRS) alone as a primary treatment option.

Methods

The present study is a retrospective series of eight patients (six men and two women) diagnosed with obstructive hydrocephalus due to brain tumors treated with radiosurgery between April 2013 and February 2021. The primary endpoint of the present study is to report our institutional experience regarding the control of symptomatic obstructive hydrocephalus due to brain tumors treated with upfront radiosurgery.

Results

The mean age was 52 years (range, 5-79). The most common presenting symptoms included headache (100%), vision-related symptoms (75%), and ataxia (37.5%). All patients showed symptom improvement after radiosurgery, five (62.5%) patients showed resolution in less than three days and the rest of the patients resolved hydrocephalus in a longer timeframe (more than three days). All patients lowered their Evans index compared to the index documented before radiosurgery, in a range from 0.02 to 0.17.

Conclusion

Radiosurgery is a non-invasive alternative treatment for primary and secondary brain tumors that debut with obstructive hydrocephalus, tumors expected to have a high alpha/beta ratio might be suitable to attempt radiosurgery to avoid permanently implanted devices such as VP shunts or other invasive procedures such as a third ventriculostomy. The present study demonstrated that in selected cases SRS can lead to hydrocephalus symptom resolution along with a decrease in ventricular size in a relatively short time frame. Little evidence exists regarding the effect of SRS on symptomatic hydrocephalus resolution and further histology-specific studies are required. We acknowledge that this approach requires immediate access to radiosurgery and close clinical follow-up to ensure success.

## Introduction

Hydrocephalus is a build-up of cerebrospinal fluid (CSF) in the brain and is characterized by abnormal dilatation of the cerebral ventricles. The CSF accumulation may be due to obstruction in the normal flow or an imbalance in production and absorption [1]. The obstruction most commonly occurs at the foramen of Monro, the aqueduct of Sylvius, the fourth ventricle, and the foramen magnum; most tumors with a significant size or that produce a mass effect by edema can impede circulation at any point of CSF pathways causing obstructive hydrocephalus [2]. Frequent tumors associated with obstructive hydrocephalus include ependymoma, subependymal giant cell astrocytoma, choroid plexus papilloma, craniopharyngioma, pituitary adenoma, hypothalamic glioma, hamartoma, and metastatic tumors. Similarly, posterior fossa tumors are a common cause. Obstructive hydrocephalus also presents in 70% of tumors located in the pineal region [3].

Patients can be either symptomatic, have symptoms related to the primary tumor, or have hydrocephalus-related symptoms [1]. Generally, symptomatic patients are candidates for VP shunt placement to reduce acute symptoms. Direct removal of the lesions, endoscopic third ventriculostomy (ETV) plus biopsy, and additional shunt procedures are the most common treatment options. The primary disease diagnosis directs the subsequent management and follow-up strategy for these patients after hydrocephalus has been resolved [4]. SRS has proven to substantially reduce tumor volume in short intervals of time in tumors that are expected to have high alpha/beta ratios [5,6]. In symptomatic large brain metastases from lung and breast histology, substantial symptom improvement or resolution can be typically seen in 72 hours in the majority of patients [5], this can also be seen in primary brain tumors with expected high alpha/beta [6]. Thus, the rationale for attempting to cure secondary hydrocephalus by quickly shrinking the tumor causing the CSF obstruction with radiosurgery might be worth exploring, with the aim to spare an invasive procedure such as a permanent implant of a VP shunt. In a previous report from our institution using two-staged radiosurgery for primary large brain tumors, two patients presented with symptomatic obstructive hydrocephalus; giving upfront radiosurgery avoided the need for shunt placement or any other invasive intervention as quick tumor response to radiosurgery was obtained thus resolving the secondary hydrocephalus [6]. We reviewed our case series of patients that presented with symptomatic hydrocephalus secondary to brain tumors suspected of having high alpha/beta ratios that were managed with upfront radiosurgery as a sole treatment alternative.

To our knowledge, this is the first study to report early improvement of symptomatic obstructive hydrocephalus associated with brain tumors using SRS. There is little evidence regarding the resolution of symptomatic hydrocephalus secondary to brain tumors using SRS [6-10]. The primary endpoint of the present study is to report our initial experience regarding the resolution of symptomatic obstructive hydrocephalus due to brain tumors treated with radiosurgery.

## Materials and methods

The present study is a retrospective series of patients diagnosed with symptomatic obstructive hydrocephalus due to brain tumors (hydrocephalus was defined as Evans index >0.30) treated with either single session or adaptive/two-session radiosurgery technique. Between April 2013 and February 2021 we reviewed patient’s clinical records that met the following criteria: patients treated with radiosurgery with either a Linear Accelerator (LINAC) (TomoTherapy Incorporated, Madison WI, USA) or a rotating gamma-ray unit (Infini, Masep Medical Company, Shenzhen, China). Both genders were included, with no age restriction for patients presenting with symptomatic obstructive hydrocephalus due to primary or secondary brain tumor documented on magnetic resonance imaging (MRI), and follow-up of at least one month with MRI after radiosurgery. Criteria for exclusion included patients with incomplete medical records, without follow-up MRI or lost to follow-up.

A set of variables were considered among all patients: for the clinical profile, we included gender, age, diagnoses, anatomic area of brain tumor, and presenting symptoms. The radiosurgical plan characteristics comprehend the date of radiosurgical treatment, type of radiosurgical plan, tumor volume, and radiation dose delivered. And for clinical evolution, we measured Evans index prior to and after radiosurgery, response evaluation criteria for solid tumors (RECIST), follow-up MRI, time of symptom resolution with a cut-off point of three days, patients’ actual status (alive or deceased), and progression-free survival.

The Evans index was calculated from the ratio of the maximum width of the frontal horns of the lateral ventricles (A) and the maximal internal diameter of the skull (B) at the same level in axial MRI, performed during the radiosurgery simulation process and from follow-up MRI (Evans index = A/B). The RECIST scale (Table 1) was obtained by measuring the percentage of tumor reduction in brain tumor volume documented during the simulation process and follow-up MRI.

**Table 1:** Response evaluation criteria for solid tumors (RECIST)

Grading	Tumor response
1	Complete response: the disappearance of all target lesions
2	Partial response: at least a 30% decrease in the sum of diameters of target lesions
3	Stable disease: Neither sufficient shrinkage to qualify for a partial response nor sufficient increase to qualify for progressive disease
4	Progressive disease: at least a 20% increase in the sum of diameters of target lesions

All cases were discussed and approved by a multidisciplinary team of radiation oncologists, medical physicists, and neurosurgeons. All patients and caregivers signed informed written consent. The study protocol was approved by the International Cancer Center Group ethical committee board. 

## Results

**Figure 1:**
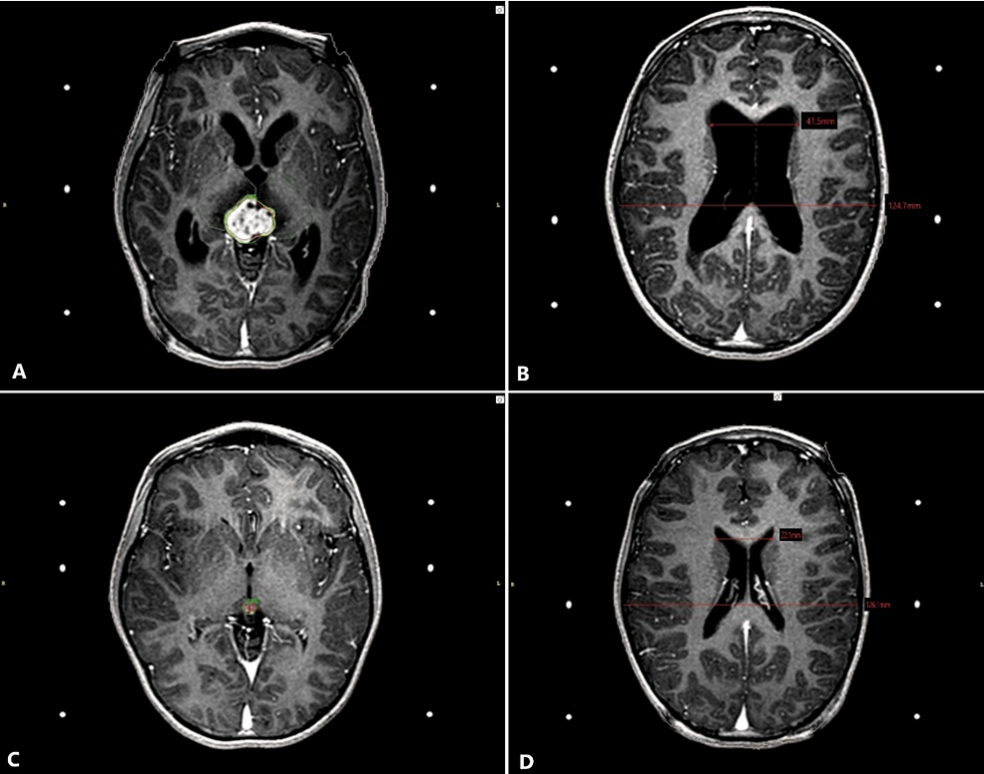
Adaptive SRS plan for patient three with pineocytoma in mesencephalic tectum causing hydrocephalus A. The first session of the adaptive SRS plan to treat a pineocytoma in the mesencephalic tectum. The plan included a GTV of six cubic centimeters (in red) with a prescription of 13 Gy with a 50% isodose line covering (in light green). The cerebral peduncles were drawn as OAR and limited the dose prescription. B. During the first session, the lateral ventricles showed an EVAN’S index of 0.33. C. After 42 days, the axial T1 gadolinium-enhanced MRI of the second session showed a reduction of the tumor in the mesencephalic tectum. The GTV (in red) included in this plan was 0.1cc and it was treated with 10 Gy with the 50% isodose line covering (in green). The tumor no longer compromises the quadrigeminal cistern. D. The lateral ventricles showed a reduction in their volume, and their EVAN’S index was 0.17. SRS: stereotactic radiosurgery; GTV: gross target volume; Gy: Grays; OAR: organs at risk.

**Figure 2:**
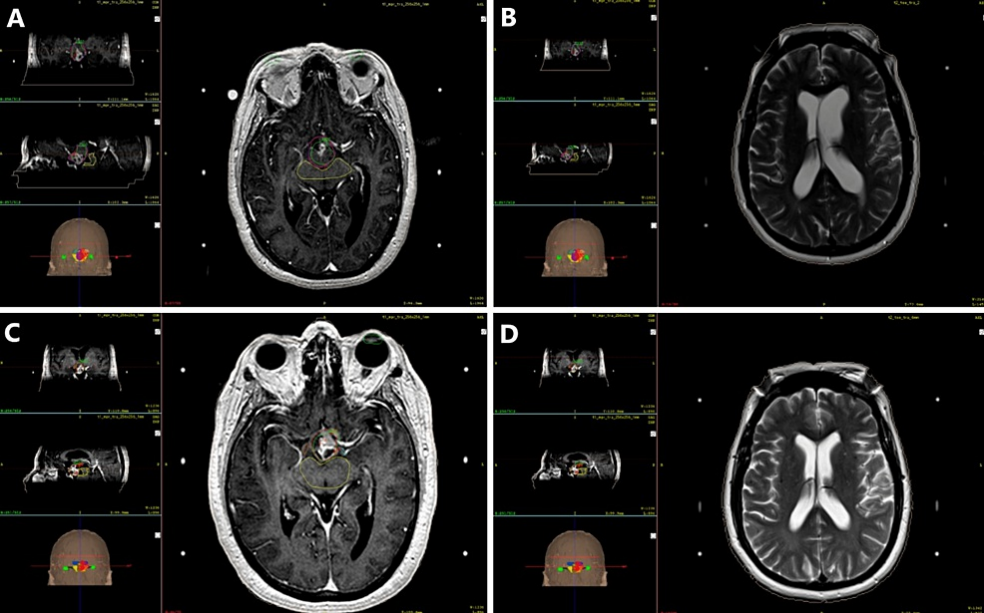
Tumor and hydrocephalus evolution for patient six Top row: prior to SRS. (A) Axial T1 gadolinium-enhanced MRI the day before treatment showed a large hypothalamic glioma. PTV in this plan (7.3 cc) was drawn within the GTV (8 cc). The prescription dose for PTV was 13 Gy at a 50% isodose curve and GTV received a median and maximum prescription dose of 0.5 and 1 Gy, respectively. (B) Axial T2 MRI on the day of treatment, the image shows dilation of the lateral ventricles, the transpendymal edema and a deviation of the septum pellucidum due to hydrocephalus. EVAN’S index at that moment was 0. 34. Bottom row: Second treatment session of two-staged radiosurgery (30 days after). (C) Axial T1 gadolinium-enhanced MRI showed a tumor volume of 4.0 cc, in this case, PTV=GTV. A tumor reduction of 45% was documented compared to the first treatment session. The prescription dose, in this case, was 10.5 Gy at a 50% isodose curve. D) Regarding hydrocephalus, the lateral ventricles were not dilated, the transependymal edema had reabsorbed and septum pellucidum showed no deviation. The patient no longer had symptoms related to hydrocephalus. EVAN’S index was 0.17. PTV: planning target volume; GTV: gross tumor volume; SRS: stereotactic radiosurgery

**Figure 3:**
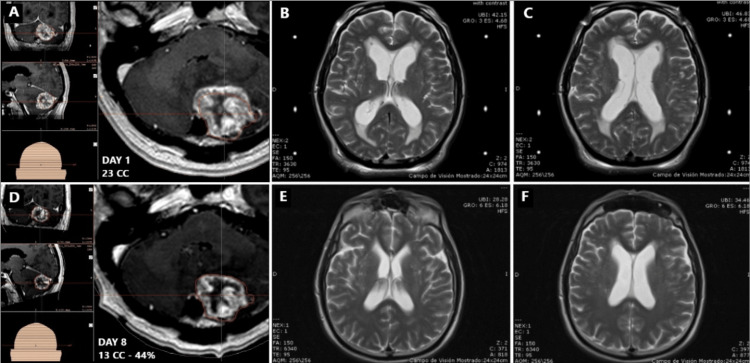
Tumor and hydrocephalus evolution for patient one Top row: prior to SRS (A) Axial T1 gadolinium-enhanced MRI the day before treatment showed large posterior fossa metastases comprising the fourth ventricle. Tumor volume at that moment was 23 cc. (B-C) Axial T2 MRI during the day of treatment. The lateral ventricles showed dilation and the transependymal edema; EVAN’S index at this moment was 0.33. Bottom row: Eight days after SRS. (D) Control axial T1 gadolinium-enhanced MRI showed 44% of its initial volume. Tumor volume eight days after SRS was 13 cc. (E-F) Eight days after SRS to the tumor, lateral ventricles did not show any dilation and the transependymal edema was reabsorbed. The patient no longer presented with symptoms related to hydrocephalus or brain metastasis. EVAN’S index was 0.27. SRS: stereotactic radiosurgery

Only eight patients met the inclusion criteria of which six (75%) were men and two (25%) were women. The mean age was 52 years (range, 5-79). Four (50%) patients had tumors in the pineal region (three pineocytomas and one germinoma) (Figure 1), two (25%) were localized in the hypothalamic area (one (12.5%) hypothalamic glioma (Figure 2), and one (12.5%) pilocytic astrocytoma). Two (25%) patients had metastasis, one located in the posterior fossa, and the other patient had the lesion at the mesencephalic tectum (Figure 3). Presenting symptoms included headache (100%), vision-related symptoms (75%), and ataxia (37.5%), among others. Table 2 shows patients' clinical profiles.

**Table 2:** Patient characteristics

Data category	N (%)
Sex	
Male	6 (75%)
Female	2 (25%)
Age; mean (range)	52 (5-79)
Diagnosis	
Brain metastasis	2 (25%)
Pineocytoma	3 (37.5%)
Germinoma	1 (12.5%)
Hypothalamic glioma	1 (12.5%)
Pilocytic astrocytoma	1 (12.5%)
Anatomic area	
Hypothalamic area	2 (25%)
Pineal region	4 (50%)
Mesencephalic tectum	1 (12.5%)
Posterior fossa (cerebellum)	1 (12.5%)
Presenting symptoms	
Headache	8 (100%)
Disorientation	2 (25%)
Ataxia	3 (37.5%)
Parinaud Syndrome	1 (12.5%)
Neck pain	2 (25%)
Paresis	1 (12.5%)
Nausea/vomiting	2 (25%)
Visual-related symptoms	6 (75%)
Radiosurgical platform	
GammaKnife (Infini)	7 (87.5%)
Linear accelerator (Tomotherapy)	1 (12.5%)

Regarding radiosurgical plan characteristics, two different types of radiosurgical platforms were used: Tomotherapy, which was used for the treatment of metastasis in one patient using a single treatment session, and Infini from which two different radiosurgical plans were used: adaptive or two-stage radiosurgery with a 30-day interval between sessions and single-session radiosurgery depending on each tumor characteristics. Table 3 shows details of radiosurgical doses and tumor volume. 

**Table 3:** Radiosurgical plan characteristics Gy: Gray; PTV: planning target volume *Two out of three patients diagnosed with pineocytoma received two-session radiosurgeries, therefore values are a mean from tumor volumes and dose delivered.

Gamma Knife (Infini)	Adaptive radiosurgery				Radiosurgery single session	
	1^st^ session dose (Gy)	Tumor volume (cc)	2^nd^ session dose (Gy)	Tumor volume (cc)	Dose (Gy)	Tumor volume (cc)
Hypothalamic glioma	13	7.3	10.5	4.1		
Germinoma	10	20.2	13	0.7		
Pineocytoma	12.5*	13.9*	11*	10.9*	12.7	12
Metastasis					12.5	25.6
Pilocytic astrocytoma					15.5	5.0
LINAC (Tomotherapy)						
Metastasis					11	PTV1: 10.1
						PTV2: 3.2

Table 4 shows a summary of key clinical data for each patient and hydrocephalus response to radiosurgery. All patients had daily follow-up for the first five days and every week thereafter to measure symptomatic response to treatment; all patients showed symptom improvement after radiosurgery, five (62.5%) patients showed resolution in less than three days and the rest of the patients resolved hydrocephalus in a longer timeframe (more than three days). The mean Evans index prior to radiosurgery was 0.35; after radiosurgery mean Evans index was 0.26 (0.09). During this three-day interval, a clinical correlation between symptom improvement and follow-up MRI was analyzed. It is worth mentioning specific cases: patient one was highly disoriented, ataxic, and had decreased strength in his lower limbs; 24 hours after radiosurgery, symptoms had improved significantly, and a follow-up MRI showed improvement in hydrocephalus. Contrarily, patient five took a longer time to recover from presenting symptoms and needed more than three days to completely resolve symptomatic hydrocephalus; the differential of Evans index in her case was only 0.02. A significant difference between these two cases is that patient five underwent ventriculostomy months prior to radiosurgery, contrary to patient one who was only treated with radiosurgery. Patients who had complete or partial tumor response (RECIST 1-2) after radiosurgery showed a reduction of Evans index below 0.31 (75% of the patients). Patients with stable disease (RECIST 3) did not lower their Evans index below 0.31. Despite this, all patients lowered their Evans index compared to the index documented before radiosurgery, in a range from 0.02 to 0.17. At the last follow-up, three patients were deceased, two from primary disease progression, and the other patient developed peritoneal tumor seeding after VP placement. The mean time of progression-free survival among these patients was 41 months (range 1-103).

**Table 4:** Summary of key clinical data for each patient and hydrocephalus response to radiosurgery RS: Radiosurgery; RECIST: response evaluation criteria for solid tumors; VP shunt: ventriculoperitoneal shunt *Patient initially was diagnosed as a pineocytoma, was further shunted for primary disease progression one year after treatment, and developed peritoneal tumor seeding

No.	Age	Sex	Primary malignancy	Radiosurgical platform	Symptoms resolution (days)	Evans score prior to RS	Evans score after RS	Evans score differential	RECIST	Patient’s status (cause of death)	Progression-free Survival (months)
1	67	M	Rectal adenocarcinoma	Infini	≤ 3	0.33	0.22	0.11	2	Deceased (Abdominal sepsis)	1
2	12	M	Germinoma*	Infini	≤ 3	0.31	0.14	0.17	1	Deceased (Peritoneal seeding)	36
3	10	M	Pineocytoma	Infini	≤ 3	0.33	0.17	0.15	1	Alive	49
4	40	M	Pineocytoma	Infini	≤ 3	0.35	0.21	0.14	2	Alive	103
5	64	F	Pineocytoma	Infini	> 3	0.49	0.47	0.02	3	Alive	57
6	72	F	Hypothalamic glioma	Infini	≤ 3	0.34	0.32	0.02	3	Alive	20
7	5	M	Pilocytic astrocytoma	Infini	> 3	0.32	0.28	0.04	2	Alive	48
8	54	F	Breast adenocarcinoma	Tomotherapy	> 3	0.33	0.29	0.04	2	Deceased (primary disease progression)	19

## Discussion

The present study, in contrast to routine neurosurgical management of secondary hydrocephalus, provides evidence of the effectiveness of SRS for the acute management of symptomatic obstructive hydrocephalus in selected cases of brain tumors with an expected high alpha/beta ratio. The linear-quadratic (LQ) model has been validated by experimental and clinical data as an equation that can aid to predict biological response after irradiation. The LQ describes the surviving fraction (SF) of clonogenic or stem cells as a function of radiation dose. The alpha and beta represent the intrinsic radiosensitivity of the cells, thus a higher alpha and beta of a tissue implies that the tissue or tumor is more sensitive to radiation, typically fast-growing tumors such as metastasis, are assigned the highest alpha-beta of ten [11]. Our previous work like those of others has demonstrated that such tumors in the majority of cases will shrink significantly in a relatively brief amount of time (days to weeks) [6]. Thus the rationale for treating secondary hydrocephalus caused by tumors known to have high alpha-beta ratio is based on the expected radiosensitivity and quick response to a high dose of radiation such as those delivered by single fraction radiosurgery with the aim of treating the cause (tumor) and not the consequence (hydrocephalus).

Comparison of the Evans index at different time frames showed a satisfactory response to treatment through the evident reduction of ventricle size by one to two months after SRS, showing no signs of recurrence until the last follow-up; patients who underwent two-staged radiosurgery showed a significant decrease in hydrocephalus by the second radiosurgical treatment (30 days after). Radiation to the tumor-causing obstruction at the third or fourth ventricle among our patients contributed to improving cerebrospinal fluid flow and resolution of hydrocephalus promptly, most tumors reduced their volume significantly in the short term. Our study findings show that SRS is an effective treatment alternative to improve symptomatic obstructive hydrocephalus in the early and acute phases of the disease and this is maintained in the long term in selected patients. The rapid response and early effect of SRS rely on the histology of the tumor and its radiosensitivity (high alpha-beta) [5,6,12-14].

A previous study reported four patients with posterior fossa metastasis causing obstructive hydrocephalus [15]. Rapid rescue radiosurgery in a three-staged session with a prescription dose ranging from 6-8.5 Gy for a 35% isodose line per treatment was performed for a period of seven days intending higher prescription dose to the central tumor [15]. All patients achieved a favorable response of obstructive hydrocephalus and positive tumor regression. Matsunaga et al. [9] reported on the effectiveness and safety of GKSRS for asymptomatic obstructive hydrocephalus associated with posterior fossa metastasis. Radiosurgical plans varied from single up to five fractions and doses ranging from 16-35 Gy depending on each tumor's characteristics. The Evans ratio at GKSRS (median 0.31) improved significantly compared with that at one-three months after GKSRS (median 0.26) and maintained at 6-12 months follow-up. All but one patient avoided surgical procedures for hydrocephalus after GKSRS. Within their findings, primary gastrointestinal cancer, single, two, and three staged radiosurgery, higher age (>65), and male sex correlated with unfavorable outcomes [9].

Among our patients, one had colorectal adenocarcinoma with metastasis to the posterior fossa, aged >65 years. Although he was programmed for adaptive radiosurgery he passed away 10 days after his first session from abdominal sepsis, nevertheless, we managed to document a radiological cure of his hydrocephalus by Day 8, which was consistent with the family report of alleviation of hydrocephalus symptoms in 48 hours after treatment and what they considered a complete resolution of symptoms on the third day. Our institutional experience with two-staged radiosurgery to large primary brain tumors [6] and brain metastasis [5] showed a mean volume reduction of 73% and 66% of lesions between sessions, respectively. As discussed in a previous study, higher intratumoral doses such as those delivered by GKRS to the lesion might correlate with prompt symptom relief including symptomatic obstructive hydrocephalus [5]; although the significance of a lower intratumoral dose variation such as those that are generally achieved with a LINAC under the same prescription dose and its effect on symptom alleviation needs to be proven. Among our patients, the only one who received SRS with LINAC, also diagnosed with brain metastasis, showed a reduction of her Evans index by 13% with a complete resolution of symptoms in less than four days and complete regression of hydrocephalus during follow-up. 

On the other hand, Gutierrez et al. [9] described a minimally invasive technique to perform a radiosurgical third ventriculostomy using LINAC in a patient with mild obstructive hydrocephalus secondary to renal cell carcinoma. One week after irradiation, the patient showed improvement in presenting symptoms including diplopia, quadriparesis, and dysphagia. CT scans did not show any changes in the metastatic lesions, even in the peritumoral edema, however, the ventricular size index (VSI) diminished by 4% (from 36% in pre radiosurgery to 32% at a week post-treatment) despite persistent aqueduct obliteration. In the follow-up head CTs, the ventricular index was maintained between 30% and 32% without further need for surgical intervention. Given the effectiveness of the procedure, the authors performed a second radiosurgical ventriculostomy recently [16]. Among our patients, the only one who received SRS with LINAC, also diagnosed with brain metastasis, showed a reduction of her Evans index by 13% with a complete resolution of symptoms in less than four days and complete regression of hydrocephalus during follow-up.

Tumors in the pineal region are usually accompanied by obstructive hydrocephalus. Common presenting symptoms among these patients include headache, gait disturbance, diplopia, and Parinaud syndrome, which is a sign of acute obstructive hydrocephalus. Li et al. [17] reported 147 cases of pineal region tumors treated with GKSRS; 16 patients within their series presented with obstructive hydrocephalus and had a VP shunt was placed to prevent perioperative and postoperative intracranial hypertension. Findings suggest that the safety of GKSRS can be improved by actively dealing with hydrocephalus prior to radiosurgical treatment. Bechri et al. published a case report on a patient with a papillary tumor of the pineal region presenting with obstructive hydrocephalus; the patient underwent ETV, which showed improvement of initial symptoms, the disappearance of nausea and vomiting, and only slight improvement in visual symptoms [18]. The tumor was treated with GK ICON with a dose of 14 Gy at a 50% isodose and the patient was followed up at three, six, and 12 months thereafter. During follow-up, the neurological examination showed progressive improvement until showing complete resolution of symptoms after radiosurgical treatment; tumor reduction and aqueduct compression disappeared after six months [18]. Zhang et al. [19] argued on the existing controversial management regarding when and how to treat hydrocephalus secondary to pineal region tumors. Authors of several clinical studies believe that VP shunt or external ventricular drainage should be performed prior to surgical intervention in order to reduce intracranial pressure and improve the general condition of patients; others believe that preoperative shunting might lead to ventricular collapse and poor tumor exposure, rendering surgical access more difficult and total tumor removal less likely [20-22]. Authors in this series suggest no preoperative VP shunting or external ventricular drainage should be performed before tumor resection unless the patient presents with a severe intracranial hypertension crisis; such decisions carry associated risks of infection and malfunction, and the incidence of shunt failure is significant [23,24]. One of our patients was initially diagnosed with pineocytoma, received radiosurgical treatment, and had a good response by improving symptoms. A year after SRS, he underwent a surgical procedure for VP shunt placement due to disease progression. He further developed peritoneal tumor seeding, which was biopsied, revealing a germinoma, ultimately causing his death.

The present study demonstrated the effectiveness of upfront SRS in symptomatic hydrocephalus secondary to brain tumors with expected high alpha-beta ratios. In our series, we presented patients with heterogeneous diagnoses with obstruction in different locations of the cerebrospinal fluid flow, all showing reduction in their Evans index. Although not all patients presented resolution of hydrocephalus (Evans index below 0.31) all of them presented a clinical resolution in a period of days to a week after SRS. Based on our findings, radiosurgery can be considered as an alternative approach instead of a VP shunt or third ventriculostomy in symptomatic hydrocephalus due to brain tumors with an expected rapid response to radiation. Of course, this requires a close follow-up of symptoms evolution, hospitalization if necessary, and immediate access to radiosurgery. Further research is necessary regarding this topic to generate safer treatment protocols for this set of patients.

## Conclusions

Upfront radiosurgery is a non-invasive alternative treatment for primary and secondary brain tumors that cause symptomatic obstructive hydrocephalus and are expected by histology to have a quick response to radiation. The present study demonstrated that immediate access to SRS leads to hydrocephalus symptom resolution along with a decrease in ventricular size in a relatively short time frame.

This is a small series and can be explained by the fact that most patients debuting with acute symptomatic hydrocephalus are usually managed by surgery either with a permanent VP shunt, upfront surgery to the tumor, or third ventriculostomy, nevertheless, this series proves that a noninvasive alternative such as radiosurgery is possible.

If upfront radiosurgery is to be undertaken, patients need to be selected based on a radiosensitive or high alpha-beta ratio, immediate access to the treatment to ensure no delay in the care of the patient and it might be necessary to consider in-hospital management until symptom resolution takes place. Little evidence exists regarding the effect of SRS on symptomatic hydrocephalus resolution and further studies are required to validate this as an alternative treatment to surgery.
